# Differentially methylated CpG sites associated with the high-risk group of prostate cancer

**DOI:** 10.1515/jib-2020-0031

**Published:** 2020-12-22

**Authors:** Anastasiya Kobelyatskaya, Elena Pudova, Maria Fedorova, Kirill Nyushko, Boris Alekseev, Andrey Kaprin, Dmitry Trofimov, Gennady Sukhikh, Anastasia Snezhkina, George Krasnov, Sergey Razin, Anna Kudryavtseva

**Affiliations:** Engelhardt Institute of Molecular Biology, Russian Academy of Sciences, Moscow, Russia, http://www.eimb.ru/; Institute of Gene Biology, Russian Academy of Sciences, Moscow, Russia, http://www.genebiology.ru/; National Medical Research Radiological Center, Ministry of Health of the Russian Federation, Moscow, Russia, https://nmicr.ru/; National Medical Research Center for Obstetrics, Gynecology and Perinatology named after Academician V.I. Kulakov, Ministry of Health of the Russian Federation, Moscow, Russia, https://en.ncagp.ru/

**Keywords:** high-risk group, methylation, prognosis, prostate cancer, TCGA, TMPRSS2-ERG

## Abstract

Prostate cancer (PC) is one of the most common and socially significant oncological diseases among men. Bioinformatic analysis of omics data allows identifying molecular genetic changes associated with the disease development, as well as markers of prognosis and response to therapy. Alterations in DNA methylation and histone modification profiles widely occur in malignant tumors. In this study, we analyzed changes in DNA methylation in three groups of PC patients based on data from The Cancer Genome Atlas project (TCGA, https://portal.gdc.cancer.gov): (1) high- and intermediate-risk of the tumor progression, (2) favorable and unfavorable prognoses within the high-risk group, and (3) TMPRSS2-ERG-positive (tumors with TMPRSS2-ERG fusion transcript) and TMPRSS2-ERG-free cases within the high-risk group. We found eight CpG sites (cg07548607, cg13533340, cg16643088, cg18467168, cg23324953, cg23753247, cg25773620, and cg27148952) hypermethylated in the high-risk group compared with the intermediate-risk group of PC. Seven differentially methylated CpG sites (cg00063748, cg06834698, cg18607127, cg25273707, cg01704198, cg02067712, and cg02157224) were associated with unfavorable prognosis within the high-risk group. Six CpG sites (cg01138171, cg14060519, cg19570244, cg24492886, cg25605277, and cg26228280) were hypomethylated in TMPRSS2-ERG-positive PC compared to TMPRSS2-ERG-negative tumors within the high-risk group. The CpG sites were localized, predominantly, in regulatory genome regions belonging to promoters of the following genes: *ARHGEF4, C6orf141, C8orf86, CLASP2, CSRNP1, GDA, GSX1, IQSEC1, MYOF, OR10A3, PLCD1, PLEC1, PRDM16, PTAFR, RP11-844P9.2, SCYL3, VPS13D, WT1*, and *ZSWIM2*. For these genes, analysis of differential expression and its correlation with CpG site methylation (*β*-value level) was also performed. In addition, STK33 and PLCD1 had similar changes in colorectal cancer. As for the CSRNP1, the ARHGEF4, and the WT1 genes, misregulated expression levels were mentioned in lung, liver, pancreatic and androgen-independent prostate cancer. The potential impact of changed methylation on the mRNA level was determined for the CSRNP1, STK33, PLCD1, ARHGEF4, WT1, SCYL3, and VPS13D genes. The above CpG sites could be considered as potential prognostic markers of the high-risk group of PC.

## 1 Introduction

Prostate cancer (PC, MeSH - D011471) is a common malignant neoplasm in men worldwide [1]. Currently, to predict the course of PC, patients are stratified into appropriate risk groups based on the following criteria: pathological stage of the tumor (pT), prostate-specific antigen (PSA) level before surgery, and Gleason score [2]. However, these criteria often incorrectly reflect the aggressive tumor phenotype. The solution to this problem can be the study of tumor molecular genetic characteristics using modern approaches. Bioinformatic analysis of omics datasets (genome, transcriptome, and methylome) enables identifying molecular changes that can be associated with the tendency of a tumor to disseminate or can predict the time from radical prostatectomy to disease progression.

Epigenetic changes occur in all types of malignant tumors and include perturbation of both the DNA methylation and the histone modification patterns [3], [4]. These changes can be associated with various clinical and pathological characteristics and, in some cases, allow to conclude about the prognosis [3]. Aberrant CpG methylation was found in various malignant tumors even at the early stages [4]. However, it is necessary to clearly distinguish between the role of aberrant methylation of the promoter regions and global hyper/hypomethylation throughout the genome, including intergenic and intronic regions. Hypermethylation of CpG islands can contribute to genetic instability and enhance cell growth, proliferation, and invasion [4]. For PC, global DNA hypomethylation is almost always associated with the late stages of the disease and is usually found in metastatic tissues [5].

The most commonly described change of the methylation pattern in PC concerns the promoter of the *GSTP1* gene [6], which is involved in DNA repair [7]. Its hypermethylation was detected in 90% of PC samples and 50% of hyperplasia prone to malignancy [8]. The *GSTP1* [9], *APC* [10], *RASSF1A* [11], *RARB* [3], *CCND2* [12], *EphA5* [13], and *PTGS2* [14] genes were detected to be hypermethylated in PC compared with adjacent normal prostate tissues. Promoter DNA methylation of *GSTP1* [15], *RARB* [16], *RASSF1* [17], and *APC* [18] was widely studied as a non-invasive marker for PC early diagnosis. Hypermethylated *GSTP1* promoter detecting in blood or urine are associated with the presence of PC [17]. Tumors carrying a mutation in the *IDH1* gene, which amount 1% of all PC cases, also have an increased level of DNA methylation [19].

In some cases, subgroups of malignant tumors are featured with the so-called CpG island methylator phenotype (CIMP) that is characterized by intense hypermethylation of the gene promoter regions and is associated with an unfavorable prognosis in colorectal cancer [20], [21]. The existence of the CIMP was firstly demonstrated for colorectal cancer and then was shown for bladder, breast, endometrial, gastric, hepatocellular, and lung cancer, as well as gliomas [21]. The presence of the TMPRSS2-ERG fusion transcript indicates one of the most common molecular subtypes of PC. The presence of this fusion transcript has been considered as a marker of unfavorable prognosis in PC [19]. CIMP has not been found in PC, however, higher overall genome methylation level was shown in the TMPRSS2-ERG-negative cases of PC [22]. It was reported that among TMPRSS2-ERG-positive samples methylation clusters were found; moreover one-third of TMPRSS2-ERG-positive samples of PC has been seen to be characterized by hypermethylated cluster [19]. However, the association of aberrant DNA methylation with the PC prognosis currently remains unclear [23].

The study aims to identify differentially methylated CpG sites associated with the high-risk group of PC, including unfavorable prognosis within the group and TMPRSS2-ERG molecular subtype, based on The Cancer Genome Atlas (TCGA) project data.

## 2 Materials and methods

### 2.1 Dataset

The present study includes PC methylation profiling data (Illumina 450k methylation arrays) and RNA-seq data from TCGA project (TCGA-PRAD) [24]. The cohort included PC patients belonging to the Caucasian population. The patients were not receiving neoadjuvant therapy. The cohort (*n* = 358) was divided into two PC groups, high (*n* = 251) and intermediate (*n* = 107) risk, according to the classification of D’Amico (Table 1) [2]. High-risk group (*n* = 251) was divided into favorable (*n* = 83) and unfavorable (*n* = 21) prognoses groups based on biochemical recurrence (postoperative PSA ≥ 0.2 ng/ml), and TMPRSS2-ERG-positive (*n* = 75) and TMPRSS2-ERG-negative (*n* = 79) groups.

**Table 1:** Clinicopathologic characteristics of the cohort.

Criteria	Parameter	High risk, *n*	Intermediate risk, *n*
Gleason score	6	8	13
	7	82	94
	8	51	–
	9	108	–
	10	2	–
Mean preoperative PSA (ng/ml)	–	13.2	6.7
Biochemical recurrence (postoperative PSA ≥ 0.2 ng/ml)	Yes	45	2
	No	183	87
Mean age (yr)	–	62	60
Pathologic tumor stage (pT)	pT2a	–	5
	pT2b	4	2
	pT2c	19	96
	pT3a	118	–
	pT3b	102	–
	pT4	7	–
Pathologic lymph nodes (pN)	pN0	172	81
	pN1	63	–
Clinical distant metastases (cM)	cM0	251	107
	cM1	–	–
Molecular subtype	1-ERG	80	34
	2-ETV1	15	6
	3-ETV4	9	3
	4-FLI1	1	1
	5-SPOP	13	7
	6-FOXA1	4	1
	7-IDH1	2	–
	8-other	35	22
Total	–	251	107

### 2.2 Methods

The analysis of differential CpG methylation was carried out in the R statistical environment (v. 3.5.2) [25]. For comparison of *β*-value between groups, BiSeq (v.1.22.0) [26] package was used. The Mann–Whitney test, *β*-regression, and logistic regression modeling were applied. We considered CpG sites (Illumina CpG IDs – cg#) with *p*-value <0.05 in all three tests as differentially methylated. To retrieve CpG sites mostly differentiating two patient groups, fold-change (Log_2_FC) and Δ*β*-value between comparison groups were calculated. Spearman’s rank correlation (standart “cor.test” function) analysis of detected CpG sites with the high-risk group was fulfilled. CpG site annotation (genomic position, gene name, promoter or enhancer) was accomplished by Ensembl [27] and GeneHancer [28] databases, UCSC browser [29], and annotatr (v.1.8.0) [30]. When selecting top-ranked CpG sites the preference was given to ones located in regulatory genomic regions (promoters or enhancers).

Differential expression analysis was carried out on the same samples using edgeR package (v.3.24.3) [31]. The trimmed mean of M-values (TMM) normalization method of count matrix was used; Quasi-likelihood (QLF), Exact Fisher’s (ET), and Mann–Whitney tests were applied for detecting differences between comparison groups. In addition, changes in gene expression level between the comparison groups (Log_2_FC) and overall gene expression level in the cohort (Log_2_CPM) were calculated. Spearman’s rank correlation (standart “cor.test” function) analysis of identified CpG sites with their gene expression level was fulfilled. Differentially expressed genes were annotated by biomaRt package (v.2.38.0) [32], [33].

## 3 Results

### 3.1 Differentially methylated CpG sites associated with the high-risk group of PC

**Figure 1:**
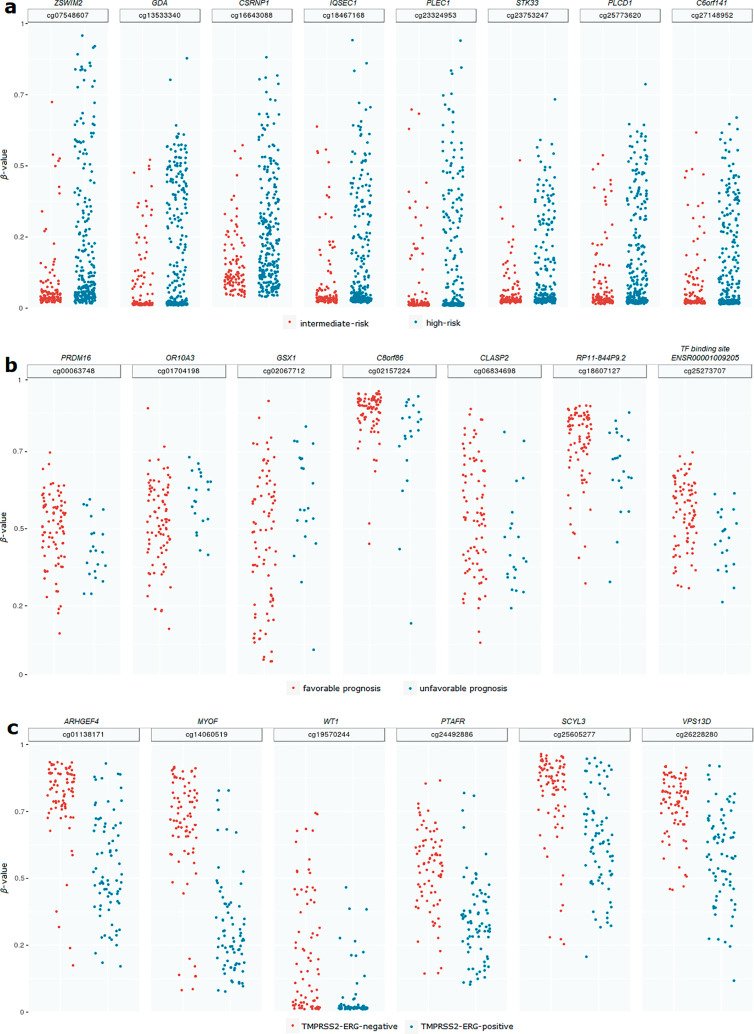
Manhattan plot of methylation level (*β*-value) of detected CpG sites among the studied groups of PC. (a) Differentially methylated CpG sites associated with the high-risk group of PC. (b) Differentially methylated CpG sites associated with the unfavorable prognosis within the high-risk group of PC. (c) Differentially methylated CpG sites associated the TMPRSS2-ERG molecular subtype within the high-risk group of PC.

We identified eight hypermethylated CpG sites (*p*-value ≤0.05; FC >1; Δ*β*-value >0) under comparing high and intermediate-risk groups: cg07548607, cg13533340, cg16643088, cg18467168, cg23324953, cg23753247, cg25773620, and cg27148952 (Figure 1a). These CpG sites were located in the promoters of the following genes [27], [28], [29], [30]: *ZSWIM2, GDA, CSRNP1, IQSEC1, PLEC1, STK33, PLCD1*, and *C6orf141*, respectively (Table 2).

**Table 2:** Differentially methylated CpG sites associated with the high-risk group of PC.

CpG site ID (Illumina 450k)	Position (hg19)	Gene (region)	Linear regression, *p*-value	Logistic regression, *p*-value	Mann–Whitney, *p*-value	Spearman’s correlation coefficient, *r*_*s*_	*p*-value	Δ*β*-value	FC
cg07548607	chr2: 187713964	*ZSWIM2* (promoter)	3.78E-02*	2.36E-02*	1.76E-07*	0.24	4.28e-06*	0.12	2.14
cg13533340	chr9: 74764495	*GDA* (promoter)	2.77E-02*	7.36E-03*	1.80E-04*	0.24	3.65e-06*	0.10	2.02
cg16643088	chr3: 39188743	*CSRNP1* (promoter)	1.02E-02*	6.14E-03*	2.01E-07*	0.28	8.89e-08*	0.11	1.67
cg18467168	chr3: 13114803	*IQSEC1* (promoter)	4.65E-03*	2.26E-03*	5.34E-04*	0.18	5.86e-04*	0.07	1.70
cg23324953	chr8: 145013728	*PLEC1* (promoter)	3.33E-04*	8.77E-03*	7.37E-03*	0.17	1.37e-03*	0.07	1.85
cg23753247	chr11: 8615842	*STK33* (promoter flank)	1.97E-02*	8.05E-05*	1.03E-02*	0.20	1.56e-04*	0.06	1.81
cg25773620	chr3: 38071309	*PLCD1* (promoter)	4.47E-04*	3.78E-03*	5.32E-04*	0.19	2.91e-04*	0.07	1.68
cg27148952	chr6: 49518347	*C6orf141* (promoter flank)	5.14E-03*	1.44E-02*	1.56E-02*	0.18	7.06e-04*	0.06	1.63
cg00063748	chr1: 3352986	*PRDM16* (promoter)	4.85E-02*	4.15E-02*	1.38E-02*	−0.22	2.70E-02	−0.06	−1.15
cg06834698	chr11: 7961985	*OR10A3* (promoter)	1.18E-02*	1.51E-02*	4.05E-02*	−0.19	4.36E-02	−0.09	−1.18
cg18607127	chr5: 175630310	*RP11-844P9.2* (promoter)	1.24E-02*	3.31E-02*	4.83E-03*	−0.24	1.36E-02	−0.08	−1.11
cg25273707	chr11: 76037066	TF binding site (ENSR00001009205)	4.72E-02*	4.31E-02*	1.59E-03*	−0.32	1.04E-03	−0.09	−1.18
cg01704198	chr3: 33757893	*CLASP2* (promoter)	6.15E-03*	3.25E-02*	1.03E-02*	0.24	1.35E-02	0.08	1.15
cg02067712	chr13: 28364724	*GSX1* (promoter flank)	2.49E-02*	2.94E-02*	8.70E-03*	0.25	9.61E-03	0.14	1.30
cg02157224	chr8: 38368889	*C8orf86* (promoter flank)	2.05E-02*	2.53E-02*	5.62E-04*	−0.35	2.77E-04	−0.10	−1.12
cg01138171	chr2: 131724244	*ARHGEF4* (intron)	2.25E-06*	1.73E-02*	6.71E-15*	−0.61	7.26E-17	−0.26	−1.46
cg14060519	chr10: 95222867	*MYOF* (promoter)	3.25E-02*	3.91E-02*	6.53E-16*	−0.69	2.43E-23	−0.37	−2.12
cg19570244	chr11: 32457158	*WT1* (promoter)	4.53E-02*	8.92E-05*	2.14E-10*	−0.44	1.52E-08	−0.16	−3.65
cg24492886	chr1: 28474511	*PTAFR* (promoter flank)	4.16E-03*	9.70E-03*	8.81E-12*	−0.52	4.98E-12	−0.19	−1.51
cg25605277	chr1: 169859761	*SCYL3* (promoter)	6.20E-03*	7.27E-03*	5.64E-09*	−0.44	1.25E-08	−0.17	−1.25
cg26228280	chr1: 12514410	*VPS13D* (enhancer)	2.44E-02*	1.30E-05*	1.05E-11*	−0.54	5.56E-13	−0.18	−1.31

*p-value ≤ 0.05.

The differential expression analysis showed that just *CSRNP1, STK33,* and *PLCD1* genes were significantly downregulated (*p*-value ≤0.05) in the high-risk group (Table 3). Moreover, expression levels of the *CSRNP1* and *STK33* genes negatively correlated with *β*-values of their CpG sites; Spearman’s rank correlation coefficients were −0.19 and −0.13 respectively (Table 3).

**Table 3:** Differentially expressed genes associated with the high-risk group of PC.

Gene	FC	LogCPM	Quasi-likelihood test, *p*-value	Exact Fisher’s test, *p*-value	Mann–Whitney test, *p*-value	Spearman’s correlation coefficient, *r*_*s*_	Spearman’s correlation coefficient, *p*-value
*ZSWIM2*	1.97	−4.58	1.49E-04*	2.88E-02*	6.01E-02	0.01	8.46E-01
*GDA*	1.08	1.02	7.68E-01	7.93E-01	2.20E-01	−0.19	2.90E-04*
*CSRNP1*	−1.30	6.49	1.20E-03*	7.06E-04*	2.86E-03*	−0.19	3.70E-04*
*IQSEC1*	−1.02	6.65	4.35E-01	4.44E-01	2.84E-01	0.06	2.90E-01
*PLEC1*	−1.01	6.32	5.69E-01	5.68E-01	1.98E-01	0.03	9.00E-01
*STK33*	−1.27	1.74	3.33E-03*	2.15E-03*	2.12E-04*	−0.13	1.79E-02*
*PLCD1*	−1.13	4.28	1.05E-03*	1.03E-03*	2.96E-04*	−0.09	7.69E-02
*C6orf141*	1.16	−0.95	2.74E-01	2.60E-01	7.10E-01	−0.06	2.51E-01
*PRDM16*	1.20	0.43	2.36E-01	2.19E-01	9.94E-01	0.22	2.55E-02*
*OR10A3*	−1.38	−4.14	3.51E-01	4.72E-01	2.42E-01	0.33	6.91E-04*
*CLASP2*	1.00	5.80	9.83E-01	9.71E-01	6.04E-01	0.10	3.13E-01
*GSX1*	6.15	−4.42	6.19E-08*	3.60E-03*	3.84E-01	−0.03	7.90E-01
*C8orf86*	1.40	−2.77	1.52E-01	1.30E-01	8.45E-02	−0.58	7.40E-11*
*ARHGEF4*	1.28	3.83	3.50E-03*	2.92E-03*	1.02E-03*	−0.22	6.98E-03*
*MYOF*	1.16	6.63	1.77E-01	1.61E-01	2.90E-01	−0.03	6.89E-01
*WT1*	2.68	0.55	1.32E-05*	1.63E-06*	1.13E-07*	−0.23	4.42E-03*
*PTAFR*	−1.13	2.62	2.45E-01	2.37E-01	8.62E-01	0.09	2.50E-01
*SCYL3*	1.31	4.50	1.78E-09*	9.24E-10*	5.89E-09*	−0.40	2.11E-07*
*VPS13D*	1.16	6.94	1.68E-03*	1.91E-03*	3.22E-03*	−0.25	1.56E-03*

*p-value < 0.05.

According to literature, cancer-associated hypermethylation was previously shown for the *STK33* [34], [35], [36]*, IQSEC1* [37], and *PLCD1* [38], [39], [40], [41], [42], [43] genes, however, a decrease in the expression was observed only for *IQSEC1* [37] and *PLCD1* [38], [39], [40], [41], [42], [43] (Table 4). *CSRNP1 and C6orf141* were found to be downregulated with no studied methylation status.

**Table 4:** Methylation and gene expression data reported for identified genes.

Gene	Pathology	Alteration	Relation	Reference
*CSRNP1*	Hepatocellular carcinoma	No methylation data, downregulated	Tumor progression	[44]
	Lung squamous cell carcinoma	No methylation data, downregulated	Tumor progression	[45]
*IQSEC1*	Non-small cell lung cancer	Hypermethylated, downregulated	Tumor progression	[37]
*PLEC1*	Pancreatic cancer	No methylation data, upregulated	–	[46]
*STK33*	Colorectal cancer	Hypermethylated, no expression data	Tumor progression	[34], [35]
	Head and neck cancers	Hypermethylated, no expression data	Tumor progression	[36]
*PLCD1*	Colorectal cancer	Hypermethylated, downregulated	Tumor progression	[40], [42]
	Breast cancer	Hypermethylated, downregulated	–	[38]
	Gastric cancer	Hypermethylated, downregulated	–	[39]
	Chronic myeloid leukemia	Hypermethylated, downregulated	–	[41]
	Endometrial cancer	Hypermethylated, downregulated	–	[43]
*C6orf141*	Oral squamous cell carcinoma	No methylation data, downregulated	Tumor progression	[47]
*PRDM16*	Lung cancer cell lines (A549 and HTB-182)	Hypermethylated, downregulated	–	[48]
	Non–small cell lung cancer	Hypermethylated, downregulated	–	[49]
	Gastric cancer	No methylation data, downregulated	Unfavorable prognosis	[50]
*CLASP2*	Muscle-invasive bladder urothelial cancer	No methylation data, upregulated	High-stage tumors, lymph node metastases	[51]
*ARHGEF4*	Pancreatic cancer	No methylation data, upregulated	Unfavorable prognosis	[52], [53]
*MYOF*	Pancreatic cancer	No methylation data, upregulated	Poor survival outcome	[54], [55]
	Triple-negative breast cancer	No methylation data, upregulated	Poor survival outcome	[56]
*WT1*	Prostate cancer	No methylation data, upregulated	Androgen-independent stage	[57], [58]
*PTAFR*	Breast cancer	No methylation data, Upregulated	Bone metastases	[59]

### 3.2 Differentially methylated CpG sites associated with the unfavorable prognosis in the high-risk group of PC

We identified seven differentially methylated CpG sites (*p*-value ≤0.05) in the unfavorable prognosis group of PC compared with the favorable one: cg00063748, cg06834698, cg18607127, cg25273707, cg01704198, cg02067712, and cg02157224. Among them, the cg01704198 and cg02067712 sites were hypermethylated (FC >1; Δ*β*-value >0), when other CpG sites were characterized by the hypomethylation status (FC <1; Δ*β*-value <0) (Figure 1b). Six identified CpG sites were localized in the promoter regions of the *PRDM16, OR10A3, RP11-844P9.2, CLASP2, GSX1,* and *C8orf86* genes; the cg25273707 CpG site belonged to the transcription factor (TF)-binding region (Table 2) [27], [28], [29], [30].

Differential expression analysis revealed no significant expression changes of the above genes between the unfavorable prognosis group and the favorable one within the high-risk group of PC (Table 3).

However, several studies noticed that the *PRDM16* gene was hypermethylated and downregulated in lung cancer (Table 4) [48], [49], [50]. The *CLASP2* gene showed differential expression levels in lung, gastric, and bladder cancers [51].

### 3.3 Differentially methylated CpG sites associated with the TMPRSS2-ERG molecular subtype in the high-risk group of PC

When studying the molecular subtype of TMPRSS2-ERG in the high-risk group, we identified six hypomethylated CpG sites (*p*-value ≤0.05; FC >1; Δ*β*-value >0) (cg01138171, cg14060519, cg19570244, cg24492886, cg25605277, and cg26228280) that were localized in the intron of *ARHGEF4,* and promoters of *MYOF, WT1, PTAFR, SCYL3,* and *VPS13D*, respectively (Figure 1c, Table 2) [27], [28], [29], [30].

Differential expression analysis showed that the *ARHGEF4, WT1, SCYL3,* and *VPS13D* genes were significantly upregulated (*p*-value ≤0.05) in TMPRSS2-ERG-positive tumors (Table 3). Furthermore, expression levels of the above genes negatively correlated with *β*-values of their CpG sites; Spearman’s rank correlation coefficients were −0.22, −0.23, −0.40, and −0.25 respectively (Table 3).

Presently, there are no data on the methylation status of *ARHGEF4, MYOF, WT1, PTAFR, SCYL3,* and *VPS13D* in the literature (Table 4). Nevertheless, *ARHGEF4* [52], [53]*, MYOF* [54], [55], [56]*, WT1* [57], [58]*, PTAFR* [59] were upregulated in pancreatic, breast, and prostate cancers.

## 4 Discussion

DNA methylation is one of the main mechanisms of gene expression regulation. In adult normal somatic cells, oncogene silencing is maintained by the promoter methylation, when promoter methylation of tumor suppressor genes does not occur [4]. Altered DNA methylation leads to the deregulation of gene expression patterns and disruption of crucial cellular processes, such as DNA repair, cell adhesion, cell cycle control, and apoptosis, contributing to the development of cancer [4], [60]. Cancer-associated genome-wide hypomethylation more often occurs than individual gene hypomethylation [60]. At the same time, hypermethylation can be seen in promoters of individual genes in carcinogenesis a lot [60]. In this study, we found both hypermethylation and hypomethylation of CpG sites of individual genes associated with the high-risk group of PC. Identified genes have not been previously reported as oncogenes or tumor suppressor genes.

Comparison of the high- and intermediate-risk groups of PC revealed eight hypermethylated CpG sites in promoters of different genes. The decreased expression has been found only for three out of eight genes (*CSRNP1*, *STK33*, and *PLCD1*). For these genes, we observed a negative correlation of CpG site methylation status (*β*-value levels) and expression changes. Spearman’s rank correlation coefficients were statistically significant but had low values. Thus, we can conclude that there is a tendency of the impact of these CpG site hypermethylation on the gene expression. The hypermethylation of other identified CpG sites was not associated with expression alterations of corresponding genes. Notably, aberrant methylation of the *STK33,* and *PLCD1* genes was observed in other cancers. In particular, often promoter hypermethylation of the *PLCD1* gene was shown to be associated with its downregulation in breast [38], gastric [39], and colorectal cancers [40], as well as chronic myeloid leukemia [41]. In colorectal cancer, *PLCD1* promoter hypermethylation and its decreased expression were correlated with tumor progression [42]. The hypermethylation of the *STK33* gene promoter was associated with progression of colorectal [34], [35] and head and neck cancers [36]; no data on the altered gene expression were previously reported. For *IQSEC1* gene, we did not observe a significant expression change correlated with the CpG methylation status. However, hypermethylation of the *IQSEC1* gene promoter and its downregulation was reported in lung cancer [37]. Methylation status of *CSRNP1* has not been earlier studied, however, the gene expression was decreased in hepatocellular [44] and lung cancers [45] correlating with tumor progression.

Seven differentially methylated CpG sites were found under comparison of the favorable and unfavorable prognosis within the high-risk group of PC. Additional analysis of differential expression of genes with identified CpG sites revealed no significant expression changes. Therefore, aberrant methylation of identified CpG sites does not influence the gene expression. Two genes (*PRDM16* and *CLASP2*) genes have been previously shown to be involved in cancer. Promotor hypermethylation and downregulated expression of the *PRDM16* gene was observed in lung cancer [49]. In gastric cancer, decreased *PRDM16* expression was associated with an unfavorable prognosis [50]. Methylation status of the *CLASP2* gene has not been studied; however, the gene upregulation was detected in bladder cancer [51].

The analysis of TMPRSS2-ERG-positive tumors within the high-risk group of PC revealed six hypomethylated CpG sites in different genes, among which significant upregulation was observed for *ARHGEF4, WT1, SCYL3,* and *VPS13D*. Expression changes in these genes were negatively correlated with the *β*-value levels of the identified CpG sites. Thus, hypomethylation of cg01138171, cg19570244, cg25605277, cg26228280 CpG sites can potentially upregulate the expression of the corresponding genes. In the literature, there are no data on the methylation status of the identified genes. However, the *ARHGEF4* and *WT1* genes were characterized by increased expression in pancreatic [52], [53] and prostate cancers [57], [58] that correlated with unfavorable prognosis and poor survival of patients.

Likewise our study, the STK33 and the PLCD1 genes had similar both methylation changes and expression signatures in colorectal cancer, indicating their potential effect on the gene expression. With regards to the CSRNP1, the ARHGEF4, and the WT1 genes, shifted expression were noticed in lung, liver, pancreatic and androgen-independent prostate cancer. However, methylation or expression changes in SCYL3 and VPS13D have never been marked in any cancer.

## 5 Conclusion

Thus, we found differential methylation of several CpG sites associated with the high-risk group of PC. Furthermore, aberrant methylation was related to individual CpG sites located predominantly in the gene promoter regions. CSRNP1, STK33, PLCD1, ARHGEF4, WT1, SCYL3, and VPS13D were also characterized by significant changes in the mRNA levels negatively correlated with the methylation status of identified CpG sites. Identified CpG sites could be considered as potential prognostic markers of the high-risk group of PC.
